# Supplementary material for: Different RNA Elements Control Viral Protein Synthesis in Polerovirus Isolates Evolved in Separate Geographical Regions
Source: Int J Mol Sci. 2022 Oct 19;23(20):12503. doi: 10.3390/ijms232012503 (PMC9603980; doi:10.3390/ijms232012503)
Supplement: Supplementary file 1 [file ijms-23-12503-s001.zip › ijms-1944556-supplementary.pdf]

```
3´-UTRs Mediterranean isolates

10PN_MG780352_Australia      AGAGTTCCCACTACTCAGAAGTGGCGCGGCACCGGAATACGCGGCGAAGCTCAATCCGGC    60
SQ_04_1.9_JF939814_Spain     AGAGTTCCTACTACTCTGAAGTAGCGCGGCACCGGAATACGCGGCGAAGCTCAATCCGGA    60
SQ_03_7.2_JF939812_Spain     AGAGTTCCTACTACTCTGAAGTAGCGCGGCACCGGAATACGCGGCGAAGCTCAATCCGGA    60
SQ_05_9.2_JFF939813_Spain    AGAGTTCCTACTACTCTGAAGTAGCGCGGCACCGGAATACGCGGCGAAGCTCAATCCGGA    60
X76931_France                AGAGTTCCTACTACTCTGAAGTAGCGCGGCACCGGAATACGCGGCGAAGCTCAATCCGGA    60
NC_003688_France             AGAGTTCCTACTACTCTGAAGTAGCGCGGCACCGGAATACGCGGCGAAGCTCAATCCGGA    60
                             ******** ******* ***** ************************************

10PN_MG780352_Australia      AACTCAAGAACGAGAGTATAAACGTTAACGACTCCGAAAGGATAGGCAACGAACGTTTTC    120
SQ_04_1.9_JF939814_Spain     AACTCAAGAACGAGAGTATAAACGTTAACGACTCCGAAAGGATAGGCAACGAACGTTCCC    120
SQ_03_7.2_JF939812_Spain     AACTCAAGAACGAGAGTATAAACGTTAACGACTCCGAAAGGATAGGCAACGAACGTTCCC    120
SQ_05_9.2_JFF939813_Spain    AACTCAAGAACGAGAGTATAAACGTTAACGACTCCGAAAGGATAGGCAACGAACGTTCCC    120
X76931_France                AACTCAAGAACGAGAGTATAAACGTTAACGACTCCGAAAGGATAGGCAACGAACGTTCCC    120
NC_003688_France             AACTCAAGAACGAGAGTATAAACGTTAACGACTCCGAAAGGATAGGCAACGAACGTTCCC    120
                             *********************************************************  *

10PN_MG780352_Australia      ACCCATGTGGTAAACAGGGGTATTACCCCTGGCGTTTCGGTGT  163
SQ_04_1.9_JF939814_Spain     ACTCAGT--GGAAACAGAGGGATTCCCCCTGGCGTTTCGGTGT  161
SQ_03_7.2_JF939812_Spain     ACTCAGT--GGAAACAGAGGGATTCCCCCTGGCGTTTCGGTGT  161
SQ_05_9.2_JFF939813_Spain    ACTCAGT--GGAAACAGAGGGATTCCCCCTGGCGTTTCGGTGT  161
X76931_France                ACGTAGT--GGAAACAGGGGGATTCCCCCTGGCGTTTCGGTGT  161
NC_003688_France             ACGTAGT--GGAAACAGGGGGATTCCCCCTGGCGTTTCGGTGT  161
                             **  *    * ****** ** *** ******************
```

A

B

```
3´-UTRs Asiatic isolates

R-TW82_JQ700306          TCGCTTTGTGGAGACGCGCGAGACTCCACCCGGTCTCCAGTGAACCCGACCAAATCACTG      60
C_TW20_JQ700305          GCGCTTTGTGGAGACGAGCGAGACTCCACCCGGCTTCCAGTGGGCCTGTCCAAACCACTG      60
SW64_KR231963_Korea      CCGCTTTGTGGAGACGTGCGTGACTCCATCTGGCT-CCAGTGAGCCCGTCCTAATCACTG      59
SW2_KR231961_Korea       CCGCTCTGTGGAGACGTGCGTGACTCCATCTGGCTTCCAGTGAGCCCGTCCTAATCACTG      60
SW25_KR231962_Korea      CCGCTCTGTGGAGACGTGCGTGACTCCATCTGGCTTCCAGTGAGCCCGTCCTAATCACTG      60
BEIJING_EU000535         TCGCTCTGTGGGGACAAGCGTGACTCCACCCGGCCCCCAGTGAGCCTGTCCAAATCACTG      60
FJ_GQ221223              CCGCTCTGTGGAGACGAGCGTGACTCCACCCGGCAGCCAGTGGGCCCGACCAAATCACTG      60
XINJIANG_EU636992        CCGCTCTGTGGAGACGAGCGTGACTCCACCCGGCATCCAGTGGGCCCGACCAAATCACTG      60
JAN_GQ221224             CCGCTCTGTGGAGACAAGCGTGACTCCACCCGGCATCCAGTGAGCCCGACCAAATCACTG      60
CZ_HQ439023              CCGCTCTGTGGAGACGAGCGTGACTCCACCCGGCGTCCAGTGAGCCTGACCAAATCACTG      60
                          **** ***** ***  *** ******* * **   ******   ** * ** ** *****

R-TW82_JQ700306          GGAAACATCAAGCCAAAGATGTAAAATTGGAACGACTCCGTAAGGATAGGCAACGGATGT      120
C_TW20_JQ700305          GG-AACATCAAGCCAAAGATGTAAAATTGGAACGACTCCGAAAGGATAGGCAACGAACGT      119
SW64_KR231963_Korea      AT-GACATCAAGCCAAAGATGTAAAATTGGAACGACTCCGAAAGGATAGGCAACGAACGT      118
SW2_KR231961_Korea       AT-GACATCAAGCCAAAGATGTAAAATTGGAACGACTCCGAAAGGATAGGCAACGAACGT      119
SW25_KR231962_Korea      AT-GACATCAAGCCAAAGATGTAAAATTGGAACGACTCCGAAAGGATAGGCAACGAACGT      119
BEIJING_EU000535         AT-GACATCAAGCCAAAGATGTAAAATTGGAACGACTCCGAAAGGATAGGCAACGAACGT      119
FJ_GQ221223              AT-GACATCAAGCCAAAGATGTAAAATTGGAACGACTCCGGAAGGATAGGCAACGAACGT      119
XINJIANG_EU636992        AT-GACATCAAGCCAAAGATGTAAAATTGGAACGACTCCGAAAGGATAGGCAACGAACGT      119
JAN_GQ221224             AT-GACATCAAGCCAAAGATGTAAAATTGGAACGACTCCGAAAGGATAGGCAACGAACGT      119
CZ_HQ439023              AT-GACATCAAACCAAAGATGTAAAATTGGAACGACTCCGAAAGGATAGGCAACGAACGT      119
                             ******* *************************** ************** * **

R-TW82_JQ700306          TCTCACTTCTGTGAGCACAGGGG-GACTCCCCCTGGCATTCCGGTGT    166
C_TW20_JQ700305          TCTCACTATGGTGGAAACAGGGG-TTTTCCCCCTGGCGTTTCGGTGT    165
SW64_KR231963_Korea      TACCACTTTAGTGGAAACAGGG-GGACCCCCCCTGGCGTTTCGGTGT    164
SW2_KR231961_Korea       TACCACTTTAGTGGAAACAGGGGGGACCCCCCCTGGCGTTTCGGTGT    166
SW25_KR231962_Korea      TACCACTTTAGTGGAAACAGGG-GGACCCCCCCTGGCGTTTCGGTGT    165
BEIJING_EU000535         TCCCACTTTAGTGGAAACAGGG-GGATTCCCCCTGGCGTTTCGGTGT    165
FJ_GQ221223              TCCCACCTTAGTGGAAACAGGG-GAATTCCCCCTGGCGTTTCGGTGT    165
XINJIANG_EU636992        TCCCACCTTAGTGGAAACAGGG-GGACTCCCCCTGGCATTTCGGTGT    165
JAN_GQ221224             TCCCACTTTAGTGGAGACAGGG-GGATTCCCCCTGGCGTTTCGGTGT    165
CZ_HQ439023              TCCCACCTTAGTGGAAACAGGG-GGATTCCCCCTGGCGTTTCGGTGT    165
                         *  ***    ***   ******      ********* ** ******
```

Supplementary Figure S1: Sequence conservation in the 3′-UTRs of Mediterranean and Asian CABYV isolates. Separate Clustal Omega sequence alignment of the 3′-UTRs of CABYV isolates available in Genbank belonging to the Mediterranean (**A**) or to the Asian (**B**) group. Genbank accession numbers are indicated for each isolate. Asterisks mark invariable nucleotides.

```
CLUSTAL O(1.2.4) multiple sequence alignment


JMB1_LC217994_BRASIL        ACAAAAGATACAAGCGGGTG
C_TW20_JQ700305_TAIWAN      ACAAAAGAAACAAGCGGGTG
LC472499_INDONISIA          ACAAAAGAAACAAGCGGGTG
R-TW82_JQ700306_TAIWAN      ACAAAAGAAACGAGCGGGTG
FJ_GQ221223                 ACAAAAGATACGAGCGGGTG
JAN_GQ221224                ACAAAAGATACGAGCGGGTG
CZ_HQ439023                 ACAAAAGATACGAGCGGGTG
BEIJING_EU000535            ACAAAAGATACGAGCGGGTG
XINJIANG_EU636992           ACAAAAGATACGAGCGGGTG
N_FRANCE_X76931             ACAAAAGATACGAGCGGGTG
SW64_KR231963               ACAAAAGATACGAGCGGGTG
SW2_KR231961                ACAAAAGATACGAGCGGGTG
SW25_KR231962_KOREA         ACAAAAGATACGAGCGGGTG
NW18_KR231958               ACAAAAGATACGAGCGGGTG
NW5_KR231957                ACAAAAGATACGAGCGGGTG
NW1_KR231954_KOREA          ACAAAAGATACGAGCGGGTG
HS1_KR231952_KOREA          ACAAAAGATACGAGCGGGTG
HS2_KR231953                ACAAAAGATACGAGCGGGTG
HD1_KR231950_KOREA          ACAAAAGATACGAGCGGGTG
GS6_KR231949_KOREA          ACAAAAGATACGAGCGGGTG
GM16_KR231946_KOREA         ACAAAAGATACGAGCGGGTG
CY3_KR231942_KOREA          ACAAAAGATACGAGCGGGTG
K1_LC082306                 ACAAAAGATACGAGCGGGTG
SQ_04_1.9_JF939814          ACAAAAGATACGAGCGGGTG
SQ_03_7.2_JF939812          ACAAAAGATACGAGCGGGTG
SQ_05_9.2_JFF939813         ACAAAAGATACGAGCGGGTG
BL4_MK055337                ACAAAAGATACGAGCGGGTG
M3_LC217993_BRASIL          ACAAAAGATACGAGCGGGTG
SW1(14)_KR231960            ACAAAAGATACGAGCGGGTG
SW1_KR231959                ACAAAAGATACGAGCGGGTG
NW2(14)_KR231956            ACAAAAGATACGAGCGGGTG
NW2_KR231955                ACAAAAGATACGAGCGGGTG
HD118_KR231951              ACAAAAGATACGAGCGGGTG
GS2_KR231948                ACAAAAGATACGAGCGGGTG
GS1_KR231947                ACAAAAGATACGAGCGGGTG
GM7_KR231945                ACAAAAGATACGAGCGGGTG
CY6_KR231944                ACAAAAGATACGAGCGGGTG
CY4_KR231943                ACAAAAGATACGAGCGGGTG
MY-YS10_MG257903            ACAAAAGATACGAGCGGGTG
M-CY31_MG257902             ACAAAAGATACGAGCGGGTG
M-BY1_MG257901              ACAAAAGATACGAGCGGGTG
C-HS1_MG257900              ACAAAAGATACGAGCGGGTG
C-AS1_MG257899              ACAAAAGATACGAGCGGGTG
NC_003688                   ACAAAAGATACGAGCGGGTG
SQ_05_9.2_JF939813          ACAAAAGATACGAGCGGGTG
M1_LC516688_BRASIL          ACAAAAGATACGAGCGGGTG
                            ******** ** ********
```

Supplementary Figure S2: Sequence conservation in the 5′-UTRs of all CABYV isolates. Clustal Omega sequence alignment of the 5′-UTRs of CABYV isolates available in Genbank (accession numbers indicated for each isolate). Asterisks mark invariable nucleotides. Two out of the twenty nucleotides show the same variations in 4 isolates out of nearly 50.

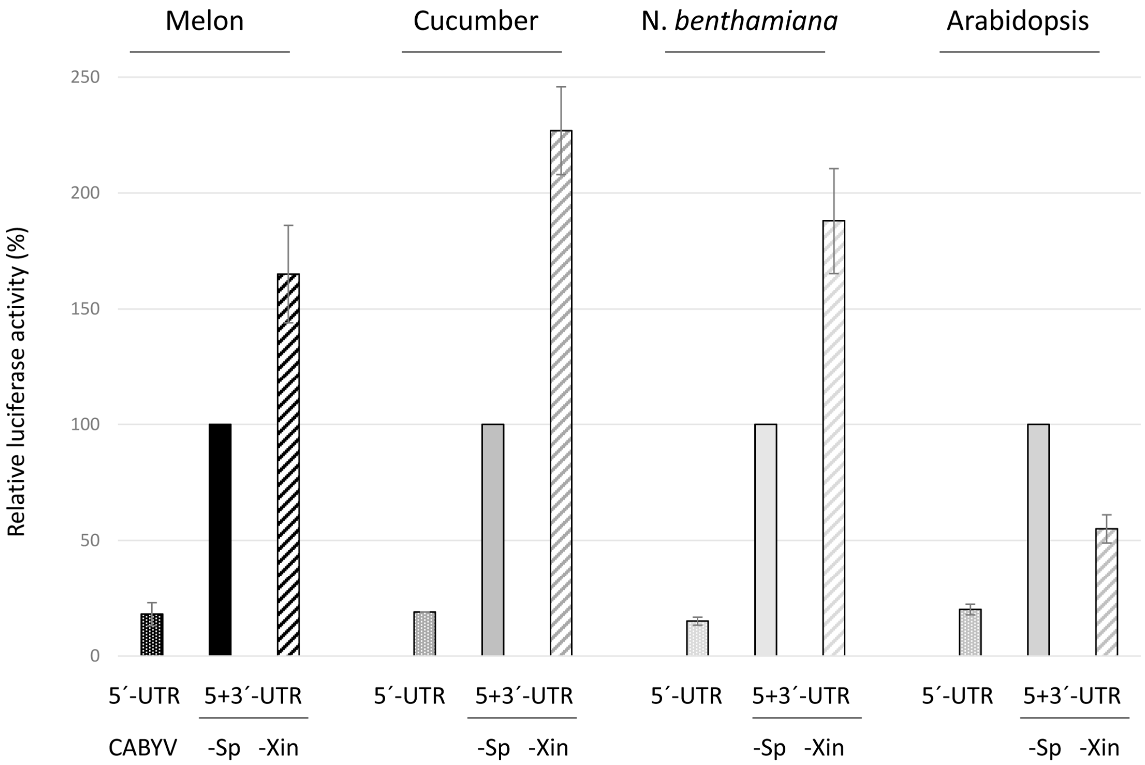

Supplementary Figure S3: Translation enhancement activities of CABYV 3´-CITEs in different host cells. *In vivo* cap-independent translation efficiency of different luc-constructs assayed in melon (columns 1-3), cucumber (columns 4-6), *N. benthamiana* (columns 7-9) and *Arabidopsis thaliana* Col1 (columns 10-12) protoplasts. Vertical columns represent measured luciferase activity (corresponding to the translation efficiency) relative to the activity obtained with the construct 5´-luc-3´-UTR of CABYV-Sp (100 %). Below the columns, the respective constructs assayed are explained. Error bars are +/- SD.

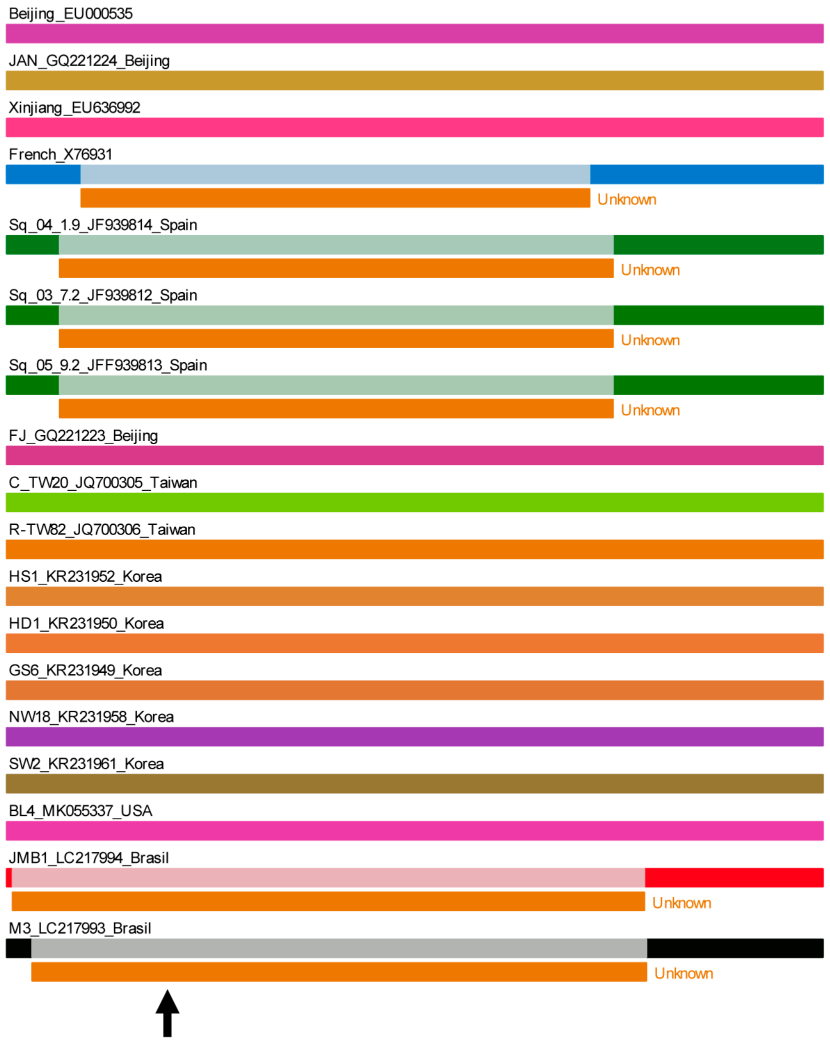

Supplementary Figure S4: Recombination prediction for 3´-ends of CABYV genomes. Recombination hypothesis generated by the RDP4 software package (https://web.cbio.uct.ac.za/~darren/rdp.html). The sequences included in this analysis performed using RDP, GENECONV, Maximum Chi-square (MaxChi), BootScan, and SisterScan (SiScan), Chimaera and 3Seq, include the last 50 nt of the ORF5 plus the following 3′-UTRs of different CABYV isolates belonging to the Mediterranean, Asian and Brazilian groups (Genbank accession number indicated for each isolate). Default settings with a Bonferroni corrected P-value cut-off of 0.01 were applied. To reduce the possibility of false detection of recombination, only recombination events supported by at least two methods were selected. RDP4 colors similar sequences with similar colors. The predicted recombination in Brazilian CABYVs starts approximately at nucleotide alignment position 2 and ends at 204 from a total of 260 alignment positions. The arrow marks the approximate start of the 3´-UR sequence. The statistical significance is very high, with a P-value < 0.00001.

A

```
SQ_04_1.9_JF939814_Spain       ------------AGAGTTCCTACTACTCTGAAGTAGCGCGGCACCGGAA-TACGCGGCGA  47
SQ_03_7.2_JF939812_Spain       ------------AGAGTTCCTACTACTCTGAAGTAGCGCGGCACCGGAA-TACGCGGCGA  47
SQ_05_9.2_JFF939813_Spain      ------------AGAGTTCCTACTACTCTGAAGTAGCGCGGCACCGGAA-TACGCGGCGA  47
JMB1_LC217994_Brasil           GGGTTAGAAGGAAAACTATAAACCACTCTGGACTTGGT-GTCTCCAGTTTTAACACTATT  59
M3_LC217993_Brasil             GGGTTAGAAGGAAAACTATAAACCACTCTGGACTTGGT-GTCTCCAGTTTTAACACTATT  59
M1_LC516688_Brasil             GGGTTAGAAGGAAAACTATAAACCACTCTGGACTTGGT-GTCTCCAGTTTTAACACTATT  59
                                           * * *    ** ****** * * *   * * ** *   **

SQ_04_1.9_JF939814_Spain       AGCTCAA----------TCCGGA------AACTCAAGAACGAGAGTATAAACGTTAACGA  91
SQ_03_7.2_JF939812_Spain       AGCTCAA----------TCCGGA------AACTCAAGAACGAGAGTATAAACGTTAACGA  91
SQ_05_9.2_JFF939813_Spain      AGCTCAA----------TCCGGA------AACTCAAGAACGAGAGTATAAACGTTAACGA  91
JMB1_LC217994_Brasil           AGATCTAGCCGTTTGGATCTAGTAAGCACCAGTCATGAACGGAAC-------GAAAAC--  110
M3_LC217993_Brasil             AGATCTAGCCGTTCGGATCTAGTAAGCACCAGTCATGAACGGAAC-------GAAAAC--  110
M1_LC516688_Brasil             AGATCTAGCCGTTCGGATCTAGTAAGCACCAGTCATGAACGGAAC-------GAAAAC--  110
                               ** ** *          **  *        * *** *****  *        *  ***

SQ_04_1.9_JF939814_Spain       CTCCGAAAGGATAGGCAACGAACGTTCCCACTCAGTGGAAACAGAG-GGATTCCCCCTGG  150
SQ_03_7.2_JF939812_Spain       CTCCGAAAGGATAGGCAACGAACGTTCCCACTCAGTGGAAACAGAG-GGATTCCCCCTGG  150
SQ_05_9.2_JFF939813_Spain      CTCCGAAAGGATAGGCAACGAACGTTCCCACTCAGTGGAAACAGAG-GGATTCCCCCTGG  150
JMB1_LC217994_Brasil           CTCACGGTGAGTAGGCAACGAACGTTCTCACATAGTGAAAACAGGGGGGACTCCCCCTGG  170
M3_LC217993_Brasil             CTCACGGTGAGTAGGCAACGAACGTTCCTACATAGTAGAAACAGG-GAGACTCTCCCTGG  169
M1_LC516688_Brasil             CTCACGGTGAGTAGGCAACGAACGTTCCTACATAGTAGAAACAGG-GAGACTCTCCCTGG  169
                               ***     *  ****************  **  ***  ******    ** ** ******

SQ_04_1.9_JF939814_Spain       CGTTTCGGTGT   161
SQ_03_7.2_JF939812_Spain       CGTTTCGGTGT   161
SQ_05_9.2_JFF939813_Spain      CGTTTCGGTGT   161
JMB1_LC217994_Brasil           CGTTTCGGTGT   181
M3_LC217993_Brasil             CGTTTCGGTGT   180
M1_LC516688_Brasil             CGTTTCGGTGT   180
                               ***********
```

B

```
JMB1_LC217994_BRASIL      --------GGGTTAGAAGGAAAACTATAAACCACTCTG----GACTTGGTGTCTCCAGTT  48
M3_LC217993_BRASIL        --------GGGTTAGAAGGAAAACTATAAACCACTCTG----GACTTGGTGTCTCCAGTT  48
M1_LC516688_BRASIL        --------GGGTTAGAAGGAAAACTATAAACCACTCTG----GACTTGGTGTCTCCAGTT  48
C_TW20_JQ700305           GCGCTTTGTGGAGACGAGCGAGACT-----CCACCCGGCTTCCAGTGGGCCTGTCCAA--  53
SW64_KR231963             CCGCTTTGTGGAGACGTGCGTGACT-----CCATCTGGC-TCCAGTGAGCCCGTCCTA--  52
BEIJING_EU000535          TCGCTCTGTGGGGACAAGCGTGACT-----CCACCCGGCCCCCAGTGAGCCTGTCCAA--  53
XINJIANG_EU636992         CCGCTCTGTGGAGACGAGCGTGACT-----CCACCCGGCATCCAGTGGGCCCGACCAA--  53
                                   **  *   *    ***     ***    *     * *  *     **

JMB1_LC217994_BRASIL      TTAACACT-ATTAGATCTAGCCGTTTGGATCTAGTAAGCACCAGTCATGAACGGAACGAA  107
M3_LC217993_BRASIL        TTAACACT-ATTAGATCTAGCCGTTCGGATCTAGTAAGCACCAGTCATGAACGGAACGAA  107
M1_LC516688_BRASIL        TTAACACT-ATTAGATCTAGCCGTTCGGATCTAGTAAGCACCAGTCATGAACGGAACGAA  107
C_TW20_JQ700305           --ACCACTGGGAACATCAAGCCAAA--GATGT-------------AAAATTGGAACGA-  94
SW64_KR231963             --ATCACTGATGACATCAAGCCAAA--GATGT-------------AAAATTGGAACGA-  93
BEIJING_EU000535          --ATCACTGATGACATCAAGCCAAA--GATGT-------------AAAATTGGAACGA-  94
XINJIANG_EU636992         --ATCACTGATGACATCAAGCCAAA--GATGT-------------AAAATTGGAACGA-  94
                            * ****    * *** ****     *** *              *  *  *******

JMB1_LC217994_BRASIL      AACCTCACGGTGAGTAGGCAACGAACGTTCTCAC-ATAGTGAAAACAGGGGGGACTCCCC  166
M3_LC217993_BRASIL        AACCTCACGGTGAGTAGGCAACGAACGTTCCTAC-ATAGTAGAAACAGG-GAGACTCTCC  165
M1_LC516688_BRASIL        AACCTCACGGTGAGTAGGCAACGAACGTTCCTAC-ATAGTAGAAACAGG-GAGACTCTCC  165
C_TW20_JQ700305           ---CTCCGAAAGGATAGGCAACGAACGTTCTCACTATGGTGGAAACAGGG-GTTTTCCCC  150
SW64_KR231963             ---CTCCGAAAGGATAGGCAACGAACGTTACCACTTTAGTGGAAACAGGG-GGACCCCCC  149
BEIJING_EU000535          ---CTCCGAAAGGATAGGCAACGAACGTTCCCACTTTAGTGGAAACAGGG-GGATTCCCC  150
XINJIANG_EU636992         ---CTCCGAAAGGATAGGCAACGAACGTTCCCACCTTAGTGGAAACAGGG-GGACTCCCC  150
                             ***     *  ***************   **  * **  *******       * **

JMB1_LC217994_BRASIL      CTGGCGTTTCGGTGT    181
M3_LC217993_BRASIL        CTGGCGTTTCGGTGT    180
M1_LC516688_BRASIL        CTGGCGTTTCGGTGT    180
C_TW20_JQ700305           CTGGCGTTTCGGTGT    165
SW64_KR231963             CTGGCGTTTCGGTGT    164
BEIJING_EU000535          CTGGCGTTTCGGTGT    165
XINJIANG_EU636992         CTGGCATTTCGGTGT    165
                          ***** *********
```

Supplementary Figure S5: Sequence comparison of the 3´-UTRs of Brazilian CABYV isolates with Mediterranean and Asian ones. Separate Clustal Omega alignments of the 3´-UTR sequences of the three Brazilian CABYV isolates with 3´-UTRs from Mediterranean isolates (**A**), or Asian (**B**) isolates. Genbank accession numbers are indicated for each isolate. Asterisks mark invariable nucleotides.

Supplementary Table S1: Sequence of assayed reporter transcripts and infectious clones.

**LUC CONSTRUCTS**	5´-UTR CABYV or plasmid *Luc* 3´-UTR/3´-CITE CABYV or plasmid
5´-UTR-*Luc*	ACAAAAGATACAAGCGGGTGCC*ATGGAA* … *CGCCGTGTAA*TTCTAGA … GGTGTGGGAGGTT
Luc-3´-UTR-Sp	GCGAATTGGG … CCCGGCCGCC*ATGGAA* … *CGCCGTGTAA*TTCTAGAAGAGTTCCTA … CCCTGGCGTTTCGGTGT
Luc-3´-UTR-Xin	GCGAATTGGG … CCCGGCCGCC*ATGGAA* … *CGCCGTGTAA*TTCTAGACCGCTCTGTG … CCCTGGCATTTCGGTGT
5´-*Luc*-3´-UTR-Sp	ACAAAAGATACAAGCGGGTGCC*ATGGAA* … *CGCCGTGTAA*TTCTAGAAGAGTTCCTA … CCCTGGCGTTTCGGTGT
5´-*Luc*-3´-ΔCITE-Sp	ACAAAAGATACAAGCGGGTGCC*ATGGAA* … *CGCCGTGTAA*TTCTAGAAACTCAAGAA … CCCTGGCGTTTCGGTGT
5´-*Luc*+CITE-Sp	ACAAAAGATACAAGCGGGTGCC*ATGGAA* … *CGCCGTGTAA*TTCTAGAAGAGTTCCTA … CGAAGCTCAATCCGGA
5´-*Luc*-3´-UTR-Xin	ACAAAAGATACAAGCGGGTGCC*ATGGAA* … *CGCCGTGTAA*TTCTAGACCGCTCTGTG … CCCTGGCATTTCGGTGT
5´-*Luc*-3´-ΔCITE-Xin	ACAAAAGATACAAGCGGGTGCC*ATGGAA* … *CGCCGTGTAA*TTCTAGAATGACATCAA … CCCTGGCATTTCGGTGT
5´-*Luc*+CITE-Xin	ACAAAAGATACAAGCGGGTGCC*ATGGAA* … *CGCCGTGTAA*TTCTAGACCGCTCTGTG … GCCCGACCAAATCACTG
5´-*Luc*+CITE-Brasil	ACAAAAGATACAAGCGGGTGCC*ATGGAA* … *CGCCGTGTAA*TTCTAGAGGGTTAGAAG … TAAGCACCAGTCATGA
**VIRUS CONSTRUCTS**	
CABYV-Sp - CMTE	ACAAAAGATACAAGCGGGTGATGCAAATTG … GATAAAACCTCCTAAAGAGTTCCTA … CCCTGGCGTTTCGGTGT
CABYV-Sp - Δ-CITE	ACAAAAGATACAAGCGGGTGATGCAAATTG … GATAAAACCTCCTAAAAACTCAAGA … CCCTGGCGTTTCGGTGT
CXTE in CABYV-Sp	ACAAAAGATACAAGCGGGTGATGCAAATTG … GATAAAACCTCCTAA*CCGCTCTGTGGAGACGAGCGTGACTCCA* *CCCGGCATCCAGTGGGCCCGACCAAATCACTG*AAACTCAAGAACGAGAGTATAA … CCCTGGCGTTTCGGTGT
